# Network-cycle motif participation is associated with individual and collective wealth in Honduran villages

**DOI:** 10.1038/s41598-025-11087-7

**Published:** 2025-07-29

**Authors:** Shivkumar Vishnempet Shridhar, Selena T. Lee, Yanick Charette, George Iosifidis, Nicholas A. Christakis

**Affiliations:** 1 Human Nature Lab, Yale University, New Haven, CT USA; 2 School of Engineering and Applied Science, Yale University, New Haven, CT USA; 3 Harvard Medical School, Harvard University, Boston, MA USA; 4 School of Social Work and Criminology, Université Laval, Quebec, QC Canada; 5 Department of Software Technology, Delft University of Technology, Delft, Netherlands; 6 Department of Sociology, Yale University, New Haven, CT USA; 7 Department of Statistics and Data Science, Yale University, New Haven, CT USA

**Keywords:** Applied mathematics, Computational science, Scientific data, Statistics, Social evolution

## Abstract

Geodesic cycles, or loops of nodes connected in a sequence within a network, are an important if under-studied network motif, and their prominence or deficiency is associated with both beneficial and detrimental properties in diverse kinds of networks. Here, we examine cycles formed by people’s reports of informal borrowing/lending and friendship ties among 22,551 rural Hondurans (in 174 isolated villages), and we explore their association with personal and community wealth across two time points. We find that cycles of different lengths (i.e., 3 or 4 ties in a loop) constitute an over-represented motif, and their quantity is strongly associated with individual wealth, i.e., richer individuals are involved in more cycles. Furthermore, we introduce a new metric of cycle composition, defined as the average of some measure (e.g., wealth) of a node’s alters in its cycles, and find that this metric outperforms cycle quantity as an indicator of both current and future wealth. A longitudinal analysis also reflects a higher participation rate in future cycles among wealthier individuals. When benchmarking cycles with eigenvector centrality, we find that cycle participation offers distinctive insights. Finally, cycle composition is a strong indicator of overall village wealth. In sum, the potential for the flow of money in a village through structural social network cycles may relate to both individual-level and village-level wealth.

**Supplementary Information:**

The online version contains supplementary material available at 10.1038/s41598-025-11087-7.

## Introduction

Network motifs in which individuals are embedded (such as triangles formed by three people) can affect the behavior and experience of individuals, as well as the dynamics of the entire network^1–3^. Among such motifs, geodesic cycles, defined as closed walks over contiguous distinct edges (of which a triangle is the simplest case)^4,5^, are of special interest as they can play an important role in a range of social, biological, economic, and technological networks. Indeed, cycles facilitate dynamic processes such as node percolation, synchronization, and epidemic spreading;^6^ are associated with increased information storage capabilities of complex networks;^7^ and are key in understanding the controllability of such systems^8^. On the other hand, many real-world networks, ranging from food web and metabolic networks to power grid and transportation networks, have been found to have fewer cycles than their randomized (null model) counterparts. Cycles in such systems amplify undesirable perturbations (e.g., oscillations), and it is hypothesized that their absence emerged through evolutionary processes favoring more stable structures^9–11^ or occurred given the prevalence of certain network features, such as node hierarchies that induce tree-like structures^12^.

The role of cycles is particularly salient in economic networks which involve the flow of credit, fiat money, or alternative currencies among economic actors. Cycles are inherent – even technically necessary – to such systems, but they can amplify equity risk contagion in interbank loan networks^13^ and hinder risk assessment as they increase uncertainty about possible defaulting equilibria;^14^ moreover, their temporal variations can herald tipping points such as the 2008 financial crisis^15^. On the other hand, cycles can improve stability of interbank networks by identifying cost-efficient bailouts^16^ and by clearing mutual obligations^17^. Such findings suggest the importance of cycles compared to other popular network-based measures, such as centrality, which capture other features of network position.

In certain economic networks, such as those arising in (digital) community currency and mutual credit systems in Sardinia^2^ (Sardex) or Kenya^18^ (Sarafu), cycles are prominent and have been associated with faster money circulation and healthier credit accounts. Their presence, therefore, beyond facilitating money flow, appears crucial both for the performance of individual firms and for the robustness of the whole system. Interestingly, even in social networks where there are no actual material or monetary exchanges, the presence of cycles has been proved theoretically, and verified empirically, to enable sustained node collaboration (exchange of favors), operationalizing, essentially, a sort of social capital^19^. Social capital has been shown to be a strong predictor of economic mobility in growing children and adolescents^20^, and socio-economic status (SES) reciprocally drives many aspects of social relationships in all age groups^21^. And cyclic interactions have also been found to play a crucial role in creating latent interactions, even when there is no material transfer^16^.

Motivated by these findings, we formulate the hypothesis that participation in cycles might be associated with upward economic mobility or monetary benefit in lower wealth groups. Therefore, we examine the role of cycles in informal peer-to-peer borrow/lend networks and also (non-monetary) friendship networks formed by 22,551 residents of 174 isolated villages in rural Honduras across two time points^22^. Such communities are primarily comprised of individuals who lack financial resources, which renders official lending institutions (e.g., banks) ineffective or prohibitively costly^23,24^. Peer-to-peer borrowing and lending based on mutual trust, therefore, can become the more common, if not the only, affordable option^24^. In such informal socio-economic systems, trust and social capital are intertwined and often shape a sense of community^25^, which, in turn, is affected by the borrow/lend interactions^15^.

We construct self-reported undirected borrow/lending and friendship networks for each of 174 villages, based on data collected with detailed sociocentric field questionnaires, and we identify the presence of cycles of different lengths. We further measure the association (through a battery of regression models) of such cycles with individual wealth and the growth in individual wealth over time, as well as with village-wide wealth. We evaluate whether cycles specifically based on the borrow-lend network are better indicators of wealth compared to cycles built based on friendship ties. We compare the relevance of cycles against other more conventional network measures, such as eigenvector centrality, while controlling for degree centrality. Finally, we propose a new measure – cycle composition (or cycle “quality”) – which, in the present case, is the average wealth of individuals participating in each cycle, to see if it can better predict individual wealth.

## Results

### Borrow-lend and friendship networks

**Fig. 1:**
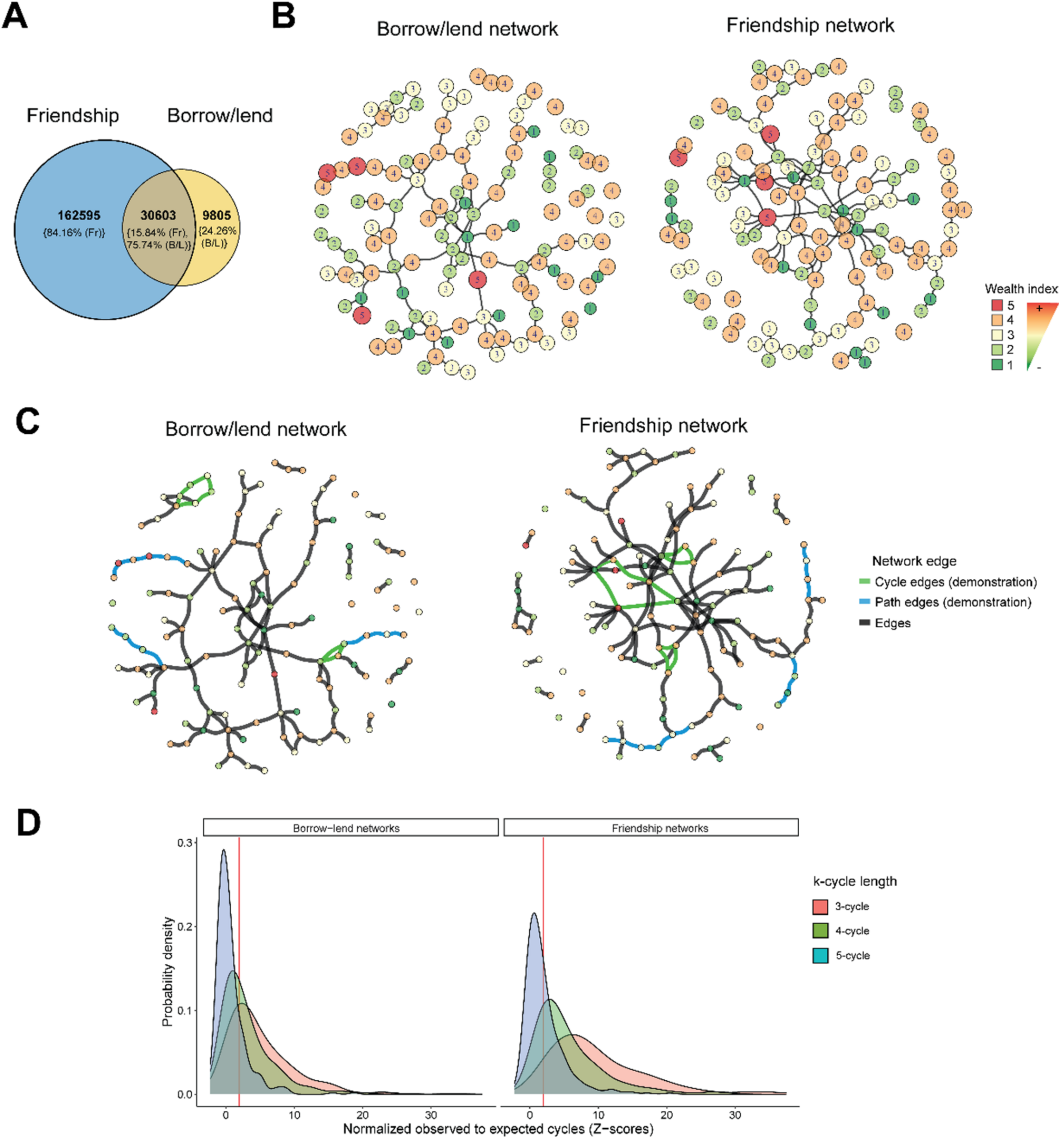
Friendship and borrow-lend networks: (**A**) Number of overlapping borrow-lend ties with friendship ties, and their share (in percentage) with respect to their categories. In other words, friendship ties (Fr) have 84.16% non-overlapping ties, while borrow-lend ties (B/L) have 24.26% non-overlapping ties. There are 30,603 ties shared between friendship and borrow-lend networks, which, occupy 15.84% and 75.74% of the total friendship and borrow-lend tie volume respectively. (**B**) Borrow/lend and friendship networks in one village colored and labelled with least wealth (1) to most wealth (5), as shown in the legend. (**C**) The same networks from panel (B) are shown here with certain selected edges highlighted to illustrate differences between paths and cycles. Green edges form cycles of lengths 3 and 5, which start and end with the same node, while blue edges indicate paths which start and end with different nodes, indicating the open-ended structure of paths (paths of length 3 and 5 shown here). (**D**) Distribution of the ratio of observed to expected cycles (expressed as Z-scores) for both borrow/lend and friendship networks for all cycle lengths (3,4,5) with colors red, green, and blue respectively, for every village (across both waves). The probability density denotes the proportion of study villages expressing a given Z-score. The red line indicates statistical significance of p-value = 0.05 (Z = 1.96). Values on the right side of the line are all statistically significant (p-value < 0.05).

In our cohort, 31.40% of all relationships are friendships (193,198/615,280 ties), even exceeding familial (17.40% or 107,058/615,280 ties) and other non-familial ties such as borrow/lend (6.56% or 40,408/615,280 ties) and making friendship ties the most dominant social interaction. However, as expected, there is a degree of overlap between the borrow/lend and friendship networks, namely 75.74% (30,603/40,408 ties) of borrow/lend ties are shared with friendship networks; however, this constitutes only 15.84% (30,603/193,198 ties) of the total friendship tie volume, which is 15.07% (30,603/202,993 ties) of the combined tie volume (borrow-lend and friendship networks) (Fig. 1A). Nevertheless, we can see from Fig. 1B that there are differences in the network structure of borrow/lend ties compared to friendship ties, in a representative village; moreover, apart from the differences in the topological position of the nodes, the two networks share only 39.90% (88/222 ties) of the ties in common.

Finally, as a comparison, we used the data to detect paths (which, unlike cycles, start and end with different individuals) of the same length as the cycles (see Fig. 1C for an illustration). Across both friendship and borrow/lend networks, we observe that the number of network cycles and paths are correlated (Figure S1). When assessing the relevance of cycles, paths of respective lengths have also been included as a control in all association models. For example, while measuring the effects of cycles of length 3, corresponding paths of length 3 were included as a covariate.

In both types of networks, we see more observed cycles than expected from simulated networks (see Methods) for all cycle lengths of ∈ {3,4,5} with a higher ratio of observed to expected cycles for smaller cycles in many villages. Cycles of length 3 had the greatest difference (borrow-lend: 72.00% (88/174) of villages; friendship: 96.28% (167/174) of villages), followed by length 4 (borrow-lend: 50.28% (87/174) of villages; friendship: 79.14% (138/174) of villages), and followed by length 5 (borrow-lend: 18.86% (33/174) of villages; friendship: 37.14% of villages) (Fig. 1D). Specifically, cycles are the dominant network motifs in the majority of villages for lengths ∈ {3,4} across both friendship (Fr) and borrow-lend networks (B/L). Overall, across all villages, each individual is part of multiple cycles of all lengths (length∈3 (µ = 1.09, SD = 2.55), 4(µ = 4.50, SD = 14.44), 5(µ = 18.55, SD = 93.06)) (Figure S2).

### Association of cycle quantity with individual wealth

**Fig. 2:**
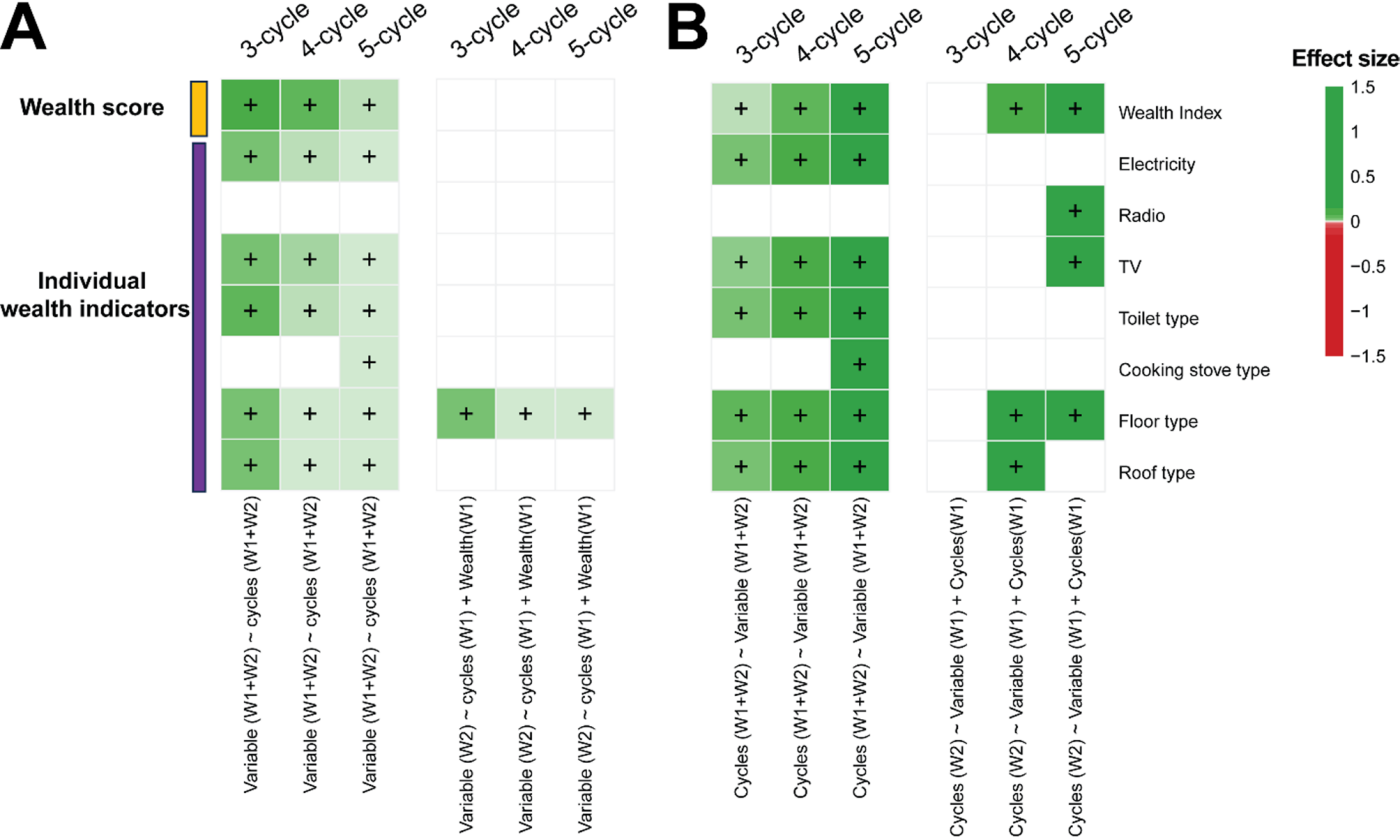
Association of cycle participation on borrow/lend networks with individual wealth: (**A**) Regression heatmap showing the association between wealth variables (in each row) and cycles (lengths ∈ {3,4,5}) for egos, along with controlling factors, for different regression models (see Methods for full model). The left 3 columns represent cross-sectional association between wealth variables and cycles, while the right 3 columns represent longitudinal relationship between cycles and subsequent wealth variables, after controlling for current wealth and other covariates, including the extent to which a node is on paths of similar length as the cycles of interest (see Methods). Green color indicates positive relationship, with the depth of color representing strength of association. Only statistically significant relationships are shown, after correcting all p-values for multiple-hypothesis testing using Benjamini-Hochberg (BH, FDR < 0.05). Analyses performed across waves are labelled as W1 (Wave 1) and W2 (Wave 2) respectively. (**B**) Similar heatmap as (**A**) with cycles as dependent variables in all the regressions shown (i.e., we swap the key dependent and independent variables).

Despite the similarities in the network characteristics, and the overlapping ties across borrow-lend and friendship relationships, we find that the number of cycles in the borrow/lend networks are better predictors of a person’s wealth compared to the number of cycles in friendship networks, after controlling for paths of similar lengths. (Figure Fig. 2A, S2).

Cycle quantity is not only associated with overall wealth index directly, but also with several individual wealth items, such as access to electricity, owning a TV set, and with the type of toilet, cooking stove, floor, or roof. To illustrate, a regression coefficient of 0.1 would mean that participating in 10 cycles (of length 5) would increase the likelihood of owning a TV set by 2.72 times (e^10*0.1^**≈**2.72). Although many of these items possess their own distinct relationship with the number of cycles in borrow/lend networks (lengths ∈ {3,4,5}), involvement in 3-cycles is in general the strongest indicator of possession of a wealth item or a higher overall wealth index (Fig. 2A).

Furthermore, richer individuals are also more likely to be involved in cycles (of borrow/lend ties) in the future (Fig. 2B). And, interestingly, higher wealth is a stronger indicator of participation in longer cycle lengths (∈ {4,5}). However, the reverse is not true, i.e., being involved in more cycles does not necessarily predict higher future wealth for these individuals.

**Fig. 3:**
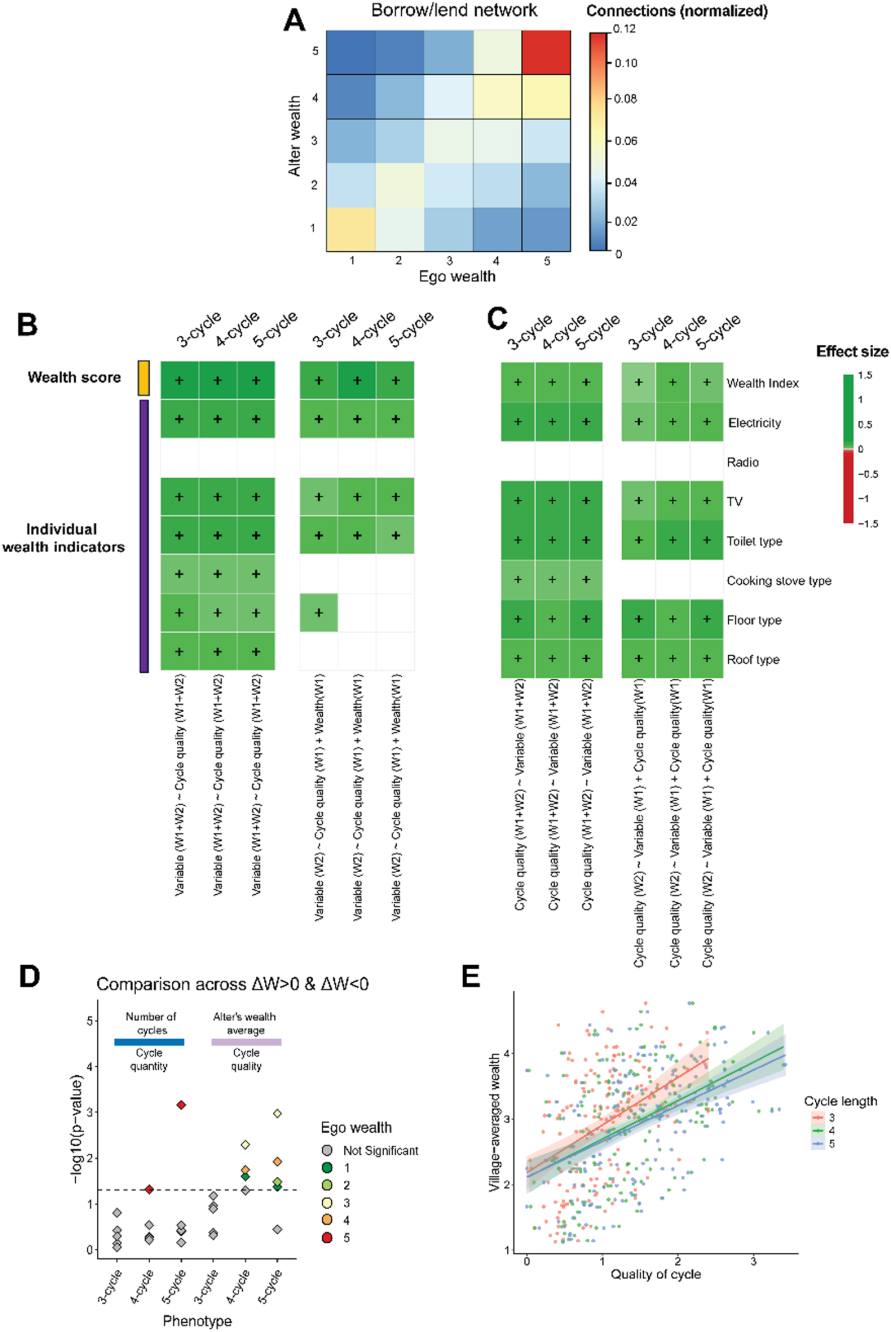
Cycle quality: (**A**) Assortativity matrix shows normalized fraction of connections between individuals belonging to all combinations of wealth classes. These stratified ties also show that richer people have richer direct connections. (**B**) Regression heatmap showing the association between wealth variables (in each row) and cycle composition (lengths ∈ {3,4,5}) for individuals, along with controlling factors (see Methods for full model). The left 3 columns represent cross-sectional association between wealth variables and cycle composition, while the right 3 columns represent longitudinal relationship between cycle composition and subsequent wealth variables after controlling for current wealth and other covariates (see Methods). Green color indicates positive relationship, with the depth of color representing strength of association. Only statistically significant relationships are shown, after correcting all p-values for multiple-hypothesis testing using Benjamini-Hochberg (BH, FDR < 0.05). Analyses performed across waves are labelled as W1 (Wave 1) and W2 (Wave 2) respectively. (**C**) Similar heatmap as (B) with network cycle quality as the dependent variables in the regressions. (**D**) Manhattan plot depicting the degree of difference in cycle quantity and composition between groups who lose and gain wealth, stratified by initial ego wealth at Wave 1. This shows that cycle quality is a significant factor in predicting wealth gain in individuals belonging to lower wealth classes (∈{1,2,3,4}). On the other hand, the number of cycles (cycle quantity) is the significant factor in wealthiest individuals (∈{5}). (**E**) Distribution of average cycle quality against average wealth across all 174 villages for cycle lengths (∈{3,4,5}). This shows a positive relationship; thick lines shown are linear fits (cycle length 3: β = 0.76, p-value = 4.28 × 10^− 12^; cycle length 4: β = 0.60, p-value = 5.82 × 10^− 13^; cycle length 5: β = 0.56, p-value = 3.55 × 10^− 13^), along with confidence intervals (shown as shaded grey areas).

On the other hand, participation in more cycles in the friendship network is not associated with wealth changes of individuals, indicating that friendship cycles are poor predictors of both wealth and wealth changes across time (Figure S3). However, networks with edges common to both borrow-lend and friendship networks show a similar association profile as pure borrow-lend networks. In addition, temporal associations with future cycles as the dependent variable show no associations, contrasting results from borrow-lend networks. Despite these differences 63.4% of the associations are present in both circumstances, which reflects the extent that borrow-lend ties overlap with friendship ties (Fig. 3A, S3).

### Benchmarking with eigenvector centrality

Since other network features, such as eigenvector centrality, can also be used to measure network position (albeit wholly dissimilar from cycles), we re-analyzed all associations after controlling for eigenvector centrality in our regression models. We found one additional association (with toilet type), with all other significant associations being analogous to our original model (Fig. 2, S4A). The reason we see such results is that we control for degree and paths in all our models, which are measures that, in turn, have a high correlation or similarity with eigenvector centrality (as shown in Figure S1).

In addition, we also show a demonstrative village (Figure S5B-C) to show how similar the popular network metrics like degree, eigenvector centrality, and betweenness centrality are to cycle centrality. We show that our model still shows significant associations after controlling for these popular network metrics, thus reinforcing the strong relationship between cycles and wealth (Figure S5A).

### Composition of cycles

Looking beyond cycle motifs, we hypothesize that it might be the properties of cycles rather than the mere number of cycles that are associated with wealth-related metrics at the individual or village level. Therefore, we introduce the metric of *cycle composition*, defined, here, as the average wealth of the nodes that are members of the cycle. Using this metric, we are interested in exploring if the composition of the cycles (cycle “quality”) a person takes part in, rather than simply the number of cycles, is associated with their wealth. Similar to Fig. 2, the same set of regression models were repeated with cycle quality to assess the overall relationship of cycle quality with ego’s wealth.

Looking at dyadic ties, it is evident from the assortativity matrix that relations between individuals tend to exhibit homophily, with borrowing and lending from people of similar wealth (Fig. 3A). When considering cycle composition, we likewise observe that richer individuals tend to participate in cycles of all lengths with other relatively wealthier individuals. Historically, homophily has been observed across race, age, gender, religion, SES, or profession, where people belonging to similar backgrounds are more likely to forge friendships leading^21,26^. We observe this behavior across borrow-lend ties as well, which could clearly be impacting the wealth and wealth flow at the individual and community level.

These associations also carry over time. Individuals participating in higher-quality cycles are more likely to gain wealth in the future (Fig. 3B). In addition, richer individuals go on to participate in higher-quality cycles over time (Fig. 3C).

In order to further understand the relative importance of these two network metrics (cycle quantity versus cycle quality), a comparative Manhattan plot shows the significance of association over all combinations of cycle lengths and stratifications of initial ego wealth (Fig. 3D). Here, cycle quality is important for wealth gain among all individuals belonging to all wealth classes (ranging from 1 to 5), while cycle quantity plays a more significant role in the wealthiest individuals (wealth class 5) for maintaining their status (Fig. 3D, S6).

### Relationship of cycles to wealth at the village level

We examined the association of cyclic motifs with the wealth of the villages as a whole. Across 174 villages, we find that bigger villages have more shorter cycles of length 3. Yet, we find that bigger villages do not necessarily have more cycles of longer lengths 4 or 5, nor do they have better-quality cycles (lengths∈{3,4,5}) than what would be expected by chance (Figure S7, S8). Looking at village wealth, wealthier villages do not have more cycles (lengths∈{3,4,5}) (Figure S9). However, wealthier villages tend to have better-quality cycles (for lengths∈{3,4,5}), even after comparing against null villages with same village size and wealth distribution (p-value < 10^− 220^ for all lengths, see Methods), indicating that cycle quality may be relevant at the community level (Fig. 3E, S10).

## Discussion

In rural settings in lower and middle income countries, trust is the foundation of social capital and, in a broader sense, of community^27,28^. Yet, borrowing from kin is shunned in many rural settings^29,30^. Therefore, many of the borrow/lend ties are built on friendship or other relationships rather than kinship. Specifically, in our Honduran cohort, we observe friendship ties as the most dominant of all social relationships, and 75.74% of borrow/lend ties overlap with friendships.

Both friendship networks and borrow/lend networks have more observed geodesic cycles of lengths 3 and 4 than what would be expected in otherwise similar randomly generated graphs. However, despite these similarities, cyclic motifs of friendship ties are not a good predictor of personal wealth or wealth gain over time. On the other hand, cycle motifs based on borrow/lend networks do have an association with wealth. Richer individuals are involved in more borrowing and lending cycles. Richer individuals are also more likely to increase their participation in more cycles in the future. However, participating in more cycles is not associated with the growth of personal wealth over time. Moreover, cycle composition (in terms of the wealth of the alters on the cycle) is a stronger indicator of current wealth and stronger predictor of future wealth. In simpler terms, any individual who gains wealth is highly likely to be involved in richer or better quality cycles. In addition, richer individuals also participate in better quality cycles in the future.

Cycle composition is also associated with community wealth, with richer villages generally having richer cycles even after controlling for the wealth of the individuals using a population permutation approach. The population-permuted null villages were considered with the same network, village size, and village wealth, while solely permuting the wealth of individuals. Community wealth is known to have an impact beyond personal financial condition, extending to healthcare infrastructure, child/maternal health, and education^31^. The participation of richer individuals in many borrow/lend cycles improves the quality of these network motifs, which might lead to village residents taking part in these richer cycles to become wealthier themselves, thus in turn helping to build overall community wealth. For instance, by analogy, lending within the community leads to reciprocal actions in Indian bazaars; and such actions could further improve the overall synergy within a community^32^. In addition, the presence of these cycles could also help improve the community through enhancements in social capital^33^. And cycle effects are not confined in networks where there is actual transfer of signals, commodities, or information; in fact, they can create latent interactions introduced by cycles^19^.

Apart from cycles, at the dyadic level, we also find homophily or more direct ties across individuals belonging to similar wealth classes, with the exception of wealthiest individuals sharing more ties with other classes as well. Homophilous behavior among individuals belonging to same SES classes has been well-established and shown to foster friendships in other settings^21,34,35^. These behaviors have been shown to have strong effects on upward income mobility of individuals. Also, forming friendships between low-SES and high-SES individuals may benefit low-SES individuals^21^. We observe a generalized version of this behavior in our results, where cycles involving diverse wealth classes significantly benefits lower-wealth individuals at future time points.

Other measures like network density, paths, and clustering are also good indicators of social capital and influence^36^. However, compared to these measures, cycles have been shown to be an indicator of the spread of wealth and information^37^, in addition to alleviating debt or credit in certain pockets of the network^2^. And here we find that cycles add information compared with these other measures.

Measuring the possible impact of cycles is tied, naturally, to the employed cycle-related metrics. And there are other extant metrics involving cycles. One of the first such metrics that was introduced was termed the “cyclic coefficient”^38^ and was defined as the average of the inverse size of the smallest loop that connects a node and any two of its neighbors. “Cycle ratio”^6^ estimates the importance of a node based on its participation to other nodes’ shortest cycles (the size of the shortest cycle containing the node). The metrics of k-cycle counts, which we use here, and the related concept of k-cycle density, is one we previously introduced to quantify important nodes and assess the prevalence (or not) of cycles in a graph^2^. A similar metric, the cycle-to-nodes-ratio, has also been used recently^39^.

We found that cycle composition is a more relevant predictor of wealth than cycle quantity. Prior studies have also investigated cycle properties, rather than their mere number. For instance, studies of food webs^40,41^ provide evidence that edge weight mixing in cycles affects network stability. From a different point of view, cycles can be seen as improving fault tolerance and robustness with respect to connectivity of two nodes (by adding redundancy). Our findings point to possible directions for increasing cycles in such social-based cooperation networks or as methods for strengthening community. Previously, interventions for increasing cycles have been proposed as a method for improving robustness^42,43^.

Our work has limitations. Since we investigated borrow-lend ties which are based on trust, we used undirected edges in all our analyses. Due to this, some cycles may be indicative of re-direction rather than circulation. This limits interpretability of these models for flow of actual wealth and economic directional analysis. Moreover, we have two time points in our analyses, which show changes across time. However,this limits the causal interpretability of cycles in its relationship with individual wealth.

In sum, we find that a network structural motif that might provide the infrastructure for the flow of resources is associated with the economic performance of individuals and villages. Cycles may be representative of trust within a group, where the trust inherent in any given individual’s borrow-lend interaction is able to be carried through a cyclical chain of such interactions. Unsurprisingly, the baseline wealth of individuals is also important, affecting the likelihood of engagement in cycles and the wealth of cycle alters. However, we also observe opportunities for economic mobility among individuals participating in cycles whose members cut across wealth classes. These findings support a bilateral relationship between social connection and economic health, while offering fertile ground for potential interventions in the future—leveraging social connection and community trust for collective economic growth.

## Methods

### Social networks construction

Village networks were constructed based on detailed hour-long surveys along with photographic census of all 24,702 adults and adolescents (age > 15) across 176 villages. Two villages were dropped due to absence of follow-up wave (Wave 2), and 2,151 individuals were excluded due to incomplete wealth or other covariate information. Overall, the analysis was performed on 22,551 individuals across 174 isolated villages. Using Trellis software^22,44,45^, name generators were asked of respondents to elicit the names of family members, friends, borrow/lend partners, and other sorts of perceived relationships. Individuals were surveyed across two waves between 2015 and 2019 (Wave 1 and Wave 2).

The Yale Institutional Review Board and the Honduran Ministry of Health approved all data collection procedures (Protocol #1506016012). All participants provided informed consent before enrolling in the study. Also, all our methods are approved and performed under guidelines by Yale Committee on Human Subjects.

All methods were performed in accordance with relevant guidelines and regulations.

The area of Western Honduras, where the study was conducted, is isolated. Over the years, as we built the data collection infrastructure in the region, we developed stronger and deeper ties to the local community, village leaders, and local health clinics. As a result of these ties, we share our results directly with our participants and other constituents at the completion of our various projects.

Both the friendship and borrow/lending networks are constructed as undirected networks. For the former, as is standard, we merged information from 3 name generators (close friend, free time, and personal and private matters; see Supplementary Table 1) and connected two individuals if at least one of them designated the other in one of those questions. For the borrow/lending network, a tie is created between two individuals if either designates the other as someone whom they trust to borrow or lend money. In both types of networks, ties among household members or to nodes with incomplete information were removed. The resulting networks included 193,198 ties for the friendship networks and 40,408 ties for the borrow/lend networks across both waves after considering individuals. Across all villages, 18,198 individuals had at least 1 friendship tie, and 17,777 individuals had at least 1 borrow-lend tie. Overall analysis was performed on all 22,551 individuals regardless of whether they had a friendship or borrow-lend tie.

### Cycle/path construction and cycle quality

Following previous modeling approaches^2,46^ we define a path $\:{p}_{uv}$ of length $\:k\:$as an ordered set of nodes $\:{p}_{uv}=\left\{u={u}_{1},\:{u}_{2},\:\dots\:,\:{u}_{k+1\:}=v\right\}\:$such that successive nodes belong to the set of network edges, i.e., $\:\left({u}_{i},{u}_{i+1}\right)\in\:E$. Also, we define a cycle $\:{c}_{uv\:}$as a path where the first and last node coincide (are identical). We define the set of all cycles of length k, of which node n is a member, as follows:

$$\:{P}_{k}\left(n\right)=\{{c}_{uv}:n\in\:\:{c}_{uv},\:u\in\:\text{N},\:\left|{c}_{uv}\right|=k\}$$

and the set of all cycles of length k in the network graph as:

$$\:{P}_{k}\left(G\right)=\{{c}_{uv}:u\in\:\text{N},\left|{c}_{uv}\right|=\text{k}\}$$

We use the k-cycle metric from previous work^2^, which is defined as the number of network cycles of length k that a node belongs to:

$$\:{C}_{{y}_{k}}=\left|{P}_{k}\right(n\left)\right|$$

We also use the k-cyclic density of the network^2^, which is defined as the logarithm of the fraction of observed cycles of length k over the expected number of cycles in a reference graph (null model):

$$\:{C}_{k}\left(G\right)=\text{l}\text{o}\text{g}\left[\frac{\left|{P}_{k}\right(G\left)\right|}{\text{m}\text{a}\text{x}\{1,\text{E}(\left|{P}_{k}\right({G}_{R}\left)\right|\left)\right\}}\right]$$

The overall cyclic density is defined as the average of the densities for the different cycle lengths i.e., from k = 3, up to k = K (in this study, K = 5), namely:

$$\:C\left(G\right)=\:\frac{1}{K}\sum\:_{k=3}^{K}{C}_{k}\left(G\right)$$

Finally, we define the new metric of “k-cycle composition” of a node n, as the average wealth of the alters that participate in its cycles, i.e.,:

$$\:{C}_{{q}_{k}}\left(n\right)=\frac{1}{k.\left|{P}_{k}\right(n\left)\right|}\sum\:_{j\in\:{P}_{k}\left(\text{n}\right)}{w}_{j}$$

Where $\:{P}_{k}$(n) is the set of cycles of length k in which node n participates (hence, it is a set of sets), |$\:{P}_{k}$(n)| is the cardinality of this set, hence the number of cycles of length k in which n participates; and $\:{P}_{k}$(n) includes as elements all the alters of n in the k-cycles (excluding n).

Networks were initially constructed using ‘igraph’ (v 2.0.3) in R. After this, cycles for each network were constructed using the kcycle.census function from the the ‘sna’ package (v 2.7-2). Similarly, paths were constructed using the kpath.census function from the ‘sna’ package.

### Expected cycles and paths per village

For calculating the expected cycles per village (as used in Fig. 1D), we employed an empirical approach previously proposed^2^ where we generated a series of null model graphs constrained by the same number of nodes, number of edges, and degree distribution as the observed village network^10,47^. We simulated 50 villages with shuffled ties per observed village in our data across both waves (Wave 1 and 2), which we used to calculate an average number of cycles and paths expected per village. Z-scores of observed (from real villages) to expected (from simulated villages) were calculated accordingly. We estimated a measure of *cyclic density* for each village, taking the natural logarithm of the ratio between observed and expected number of cycles. Likewise, we estimated a measure of *path density* for each village, taking the natural logarithm of the ratio between observed and expected number of paths.

### Wealth index construction

Household income or consumption expenditures are generally considered the ideal measurement tool of household wealth in our setting. However, consumption measures can be imprecise due to difficulties in recalling all expenditures over the past week or month^48^. Furthermore, there is an important distinction between the measurement of income and wealth, as income does not always capture accumulated financial stability in the way that wealth does, with implications for long-term poverty alleviation. The need for measurement of wealth in rural low-income contexts has led to a proliferation of methods attempting to triangulate the wealth of individual households and villages^49^, including asset-based wealth measures. In practice, such asset-based measures are easier to implement in rural contexts, have minimal recall burden on respondents, and avoid factoring in currency, which can be affected by inflation^50^.

To generate wealth rankings of households and villages, the DHS wealth index construction guidelines were followed^51^. Household factors included in the wealth index construction for the analysis can be divided into three main categories: resources, commodities, and materials and construction. Specific household resources included in the wealth index construction were the source of water for drinking and cooking; type of toilet or toilet facility used; use of a stove or a furnace/firebox for cooking; and type of cooking fuel. Household commodities included the presence each of electricity, radio, television, cell/mobile phone, non-mobile phone. Housing materials and construction variables consisted of a separate room in the house as a kitchen; presence of windows; main floor material; main wall material; and main roof material.

Subsequently, these categorical variables were analyzed using multiple correspondence analysis (MCA) to determine the underlying relative wealth rankings of households, using coordinates from the first dimension of the analysis to order households^52^. The first dimension explained 6.7% of the household wealth variance observed (see Figure S11-12). Each household was weighted by the household size (total number of members including children) and split into quintiles to classify them into relative wealth ranking, with a value of 5 representing the highest wealth. Finally, this resulting wealth measure was also validated using the Food Security Index, which we also measured (Figure S13).

### Statistical analyses

All statistical analyses were performed in R (4.1.3). Mixed effect logistic regression models were used in all regression models at the individual level. Correction of multiple hypotheses testing (Benjamini-Hochberg) was applied when applicable with a significance of FDR (False Discovery Rate) < 0.05. All tests were two-sided.

The full regression model for cycle quantity is:

$$\:Wealth\:variable\:\sim\:{Kcycle}_{i}+\:{Path}_{i}+Degree+Village\:wealth+\left(1\right|Village\:ID)$$

Wealth variables are each of the row names in the heatmap comprising of the overall Wealth index, Electricity, Radio, TV, Toilet type, Cooking type, Floor type, Roof type. Each ‘type’ variable was categorized as high (1) or low (0). The subscript ‘i’ indicates the length of cycle/path (i ∈ {3,4,5}). Village wealth was computed, and controlled for, to exclude possible village-level wealth effects at the individual scale. Village ID was also included as a random effect since all the villages in Honduras are relatively isolated (with minimal inter-village contact), which makes them independent entities here. Regressions were computed using lmerTest package (v 3.1). The regression coefficients across all models were scaled accordingly. All regressions were performed at individual level.

In addition, temporal models were performed for dependent variables at Wave 2 (W2) in all models with wealth at Wave 1 (W1) as an additional fixed effect. All other independent variables and covariates are considered at Wave 1, unless specified otherwise.

$$\:{Wealth\:variables}_{W2}\:\sim\:{Kcycle}_{i}+\:{Path}_{i}+Degree+{Wealth}_{W1}+Village\:wealth+\left(1\right|Village\:ID)$$

All models were checked (Figure S14) and found not to have meaningful multicollinearity or heteroskedasticity.

### Reverse regression model

The reverse regression model is the same as previous model with the number of cycles being the dependent variables was also specified:

$$\begin{aligned} & \:{Kcycle}_{i}\:\sim\:Wealth\:variables+\:{Path}_{i}+Degree+Village\:wealth+\left(1\right|Village\:ID){Kcycle}_{i,W2}\:\\ & \quad \sim\:Wealth\:variables+\:{Path}_{i}+Degree+{Kcycle}_{i,W1}+Village\:wealth+(1\left|Village\:ID\right)\end{aligned}$$

### Cycle composition regression models

Cycle composition regression models have village wealth and Village ID as covariates to exclude village-level effects.

$$\:Wealth\:variable\:\sim\:{Kcycle\:quality}_{\:i}+\:Village\:wealth+\left(1\right|Village\:ID)$$

$$\:{Kcycle\:quality}_{\:i}\:\sim\:Wealth\:variable+Village\:wealth+\left(1\right|Village\:ID)$$

$$\:{Kcycle\:quality}_{i,W2}\:\sim\:Wealth\:variables+\:{Kcycle\:quality}_{i,W1}+Village\:wealth+\left(1\right|Village\:ID)$$

$$\:{Wealth\:variables}_{W2}\:\:\sim\:{Kcycle\:quality}_{i}+\:{Wealth\:variables}_{W1}+Village\:wealth+\left(1\right|Village\:ID)$$

In addition, cycle composition and quality were also stratified based on initial ego wealth at Wave 1 to assess the relative wealth gains in each group.

### Village-level regression model

Linear regressions were performed at village level with village size as controls for all 174 villages:

*Village wealth ~ Village cyclic density*_*i*_ *+ Village path density*_*i*_ *+ Village size + time + (1|Village ID)*

$$\:Village\:wealth\:\sim\:{Village\:Kcycle\:quality}_{i}+\:Village\:size$$.

### Null permuted villages for cycle quality

Borrow/Lend null permuted villages create a null distribution of villages (*N* = 1000) with the same network structures and number of individuals within a village. In order to accurately discern the effects of cycle quality, only the wealth labels assigned for each node was randomly shuffled. This permutation process was done separately for every village. The resulting 1000 villages were used as null villages for every representative village (i.e. 174 × 1000 = 174,000 null villages created). In all of the null villages, as the wealth labels are permuted, the average wealth of the village remains the same.

## Electronic supplementary material

Below is the link to the electronic supplementary material.

Supplementary Material 1

Supplementary Material 2

## Data Availability

The full datasets generated and/or analyzed during the current study is not publicly available due to several considerations including commitments to research participants and the delicate nature of health and social data in these small communities which could potentially raise privacy concerns, but they are available from the lead contact upon reasonable request. The code for replicating this analysis is available at https://github.com/human-nature-lab/K_cycle_econ_networks.
