# Supplementary material for: Network-cycle motif participation is associated with individual and collective wealth in Honduran villages
Source: Sci Rep. 2025 Jul 29;15:27680. doi: 10.1038/s41598-025-11087-7 (PMC12307972; doi:10.1038/s41598-025-11087-7)
Supplement: Supplementary file 1 — Supplementary Material 1 [file 41598_2025_11087_MOESM1_ESM.docx]

**Network-cycle motif participation is associated with individual and collective wealth in Honduran villages**

Shivkumar Vishnempet Shridhar^†^*, Selena T. Lee^†^, Yanick Charette, George Iosifidis, and Nicholas A. Christakis

**Supplementary table:**

Supplementary table 1 contains all summary statistics, such as regression coefficients, p-values, p-adjusted (FDR), and correlation values for all our models.

**Supplementary figures list:**

**Figure S1**: Correlation heatmap of all network factors.

**Figure S2**: Distribution of cycles per node for all cycle lengths

**Figure S3**: Association between wealth variables and cycles (on friendship networks)

**Figure S4:** Association between wealth variables and cycles (common to both friendship and borrow/lend networks)

**Figure S5**: Benchmarking our model with eigenvector centrality and showing relationships with other network metrics.

**Figure S6**: Cycle quality and wealth change stratified by wealth class

**Figure S7**: Village size and cycle quantity

**Figure S8**: Village size and cycle quality (Cycle composition)

**Figure S9**: Village wealth and cycle quantity (Cycle composition)

**Figure S10**: Observed vs expected effects of cycle quality on village wealth

**Figure S11**: MCA (Multiple Correspondence Analysis) components

**Figure S12**: Multiple correspondence analysis of all wealth variables for first two components

**Figure S13**: Validation of the generated household wealth index

**Figure S14**: Model characteristics for regressions on wealth


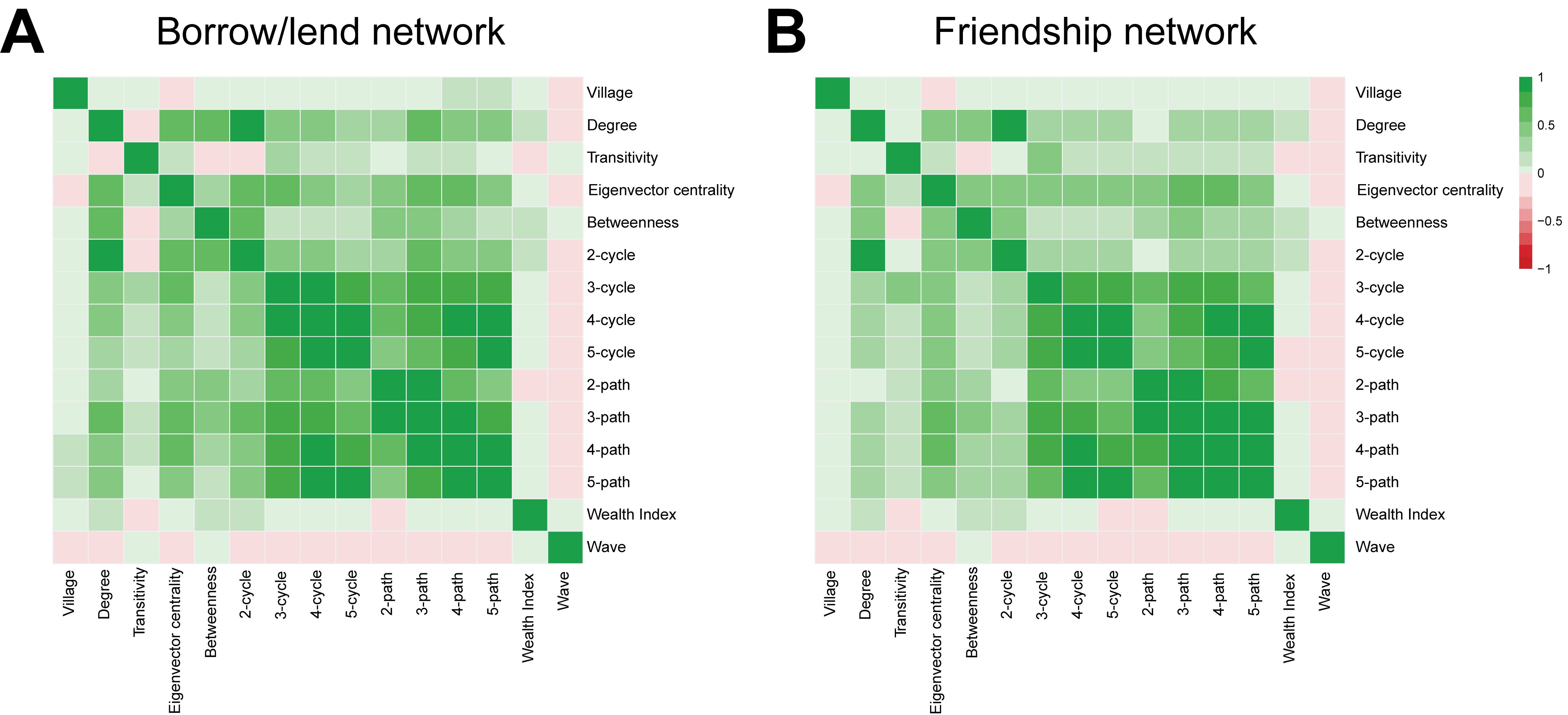


**Figure S1**: Correlation heatmap of all network factors: Correlation between all pairs of variables across all categories, including, wealth, cycles, paths, and other network factors. Pearson’s correlation was used to generate the correlation scores. Plotted heatmaps have color scales ranging from -1 (maximum negative correlation) to 1 (Maximum positive correlation). The eigenvector centrality does not exceedingly correlate with cycle centrality metrics. In particular, its correlations with the various cycle centralities are similar, and often smaller, than the correlation of those metrics with other centralities (such as the degree or transitivity). This highlights that cycle centrality can yield novel insights. Raw correlation values can be found in a Supplementary Table.


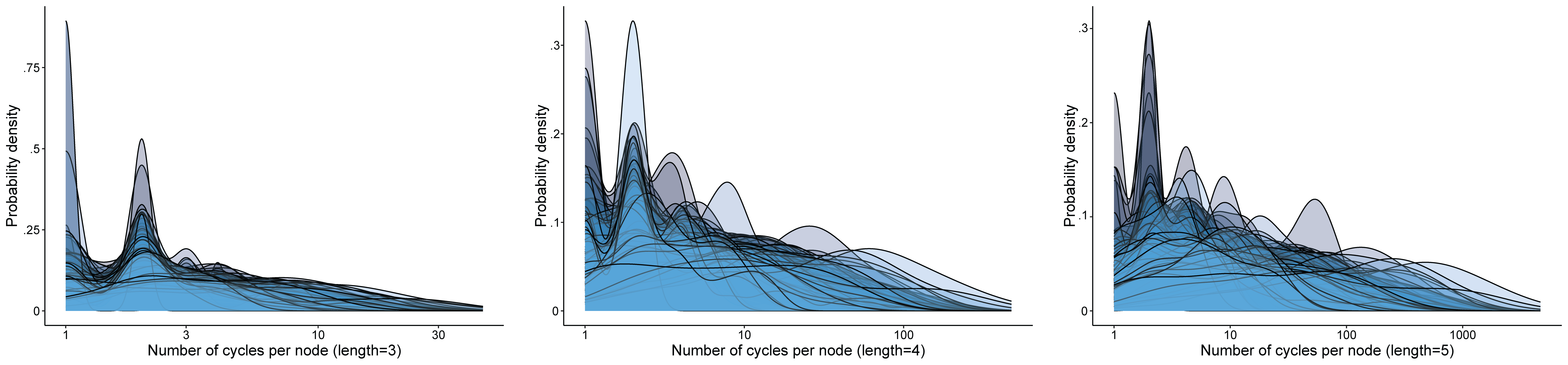


**Figure S2:** Distribution of cycles per node for all cycle lengths: Density distribution of number of cycles for every individual across the cohort, grouped by village membership. Across all villages, individuals are a part of at least 1 cycle for all lengths ∈ 3(µ=1.0864,SD=2.546), 4(µ=4.5015,SD=14.44), 5(µ=18.55,SD=93.058).


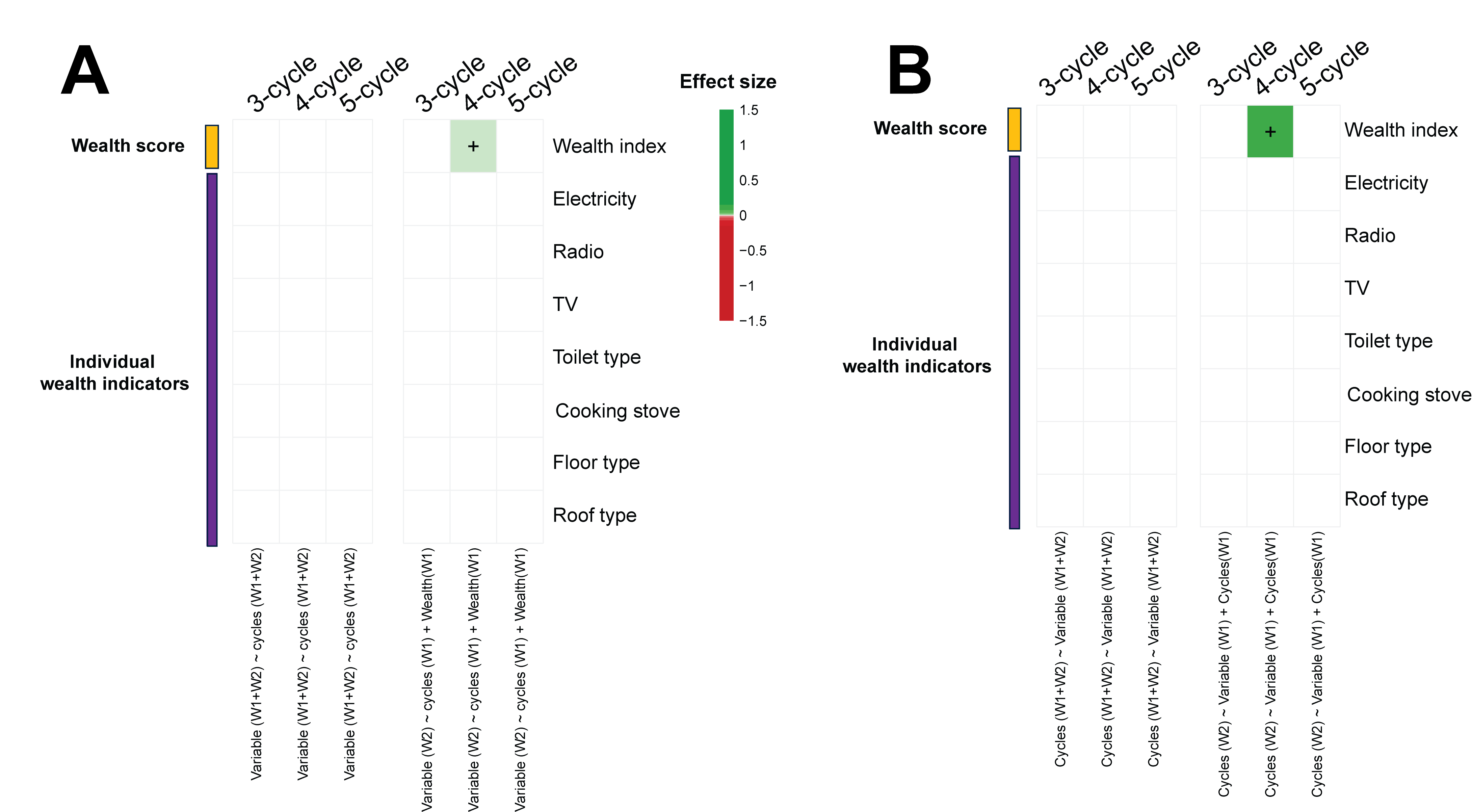


**Figure S3**: Association between wealth variables and cycles (on friendship networks): This shows only two significant relationships across both models with wealth and cycles as predictors, respectively. All associations shown are adjusted for multiple hypothesis testing using Benjamini-Hochberg correction (FDR<0.05).


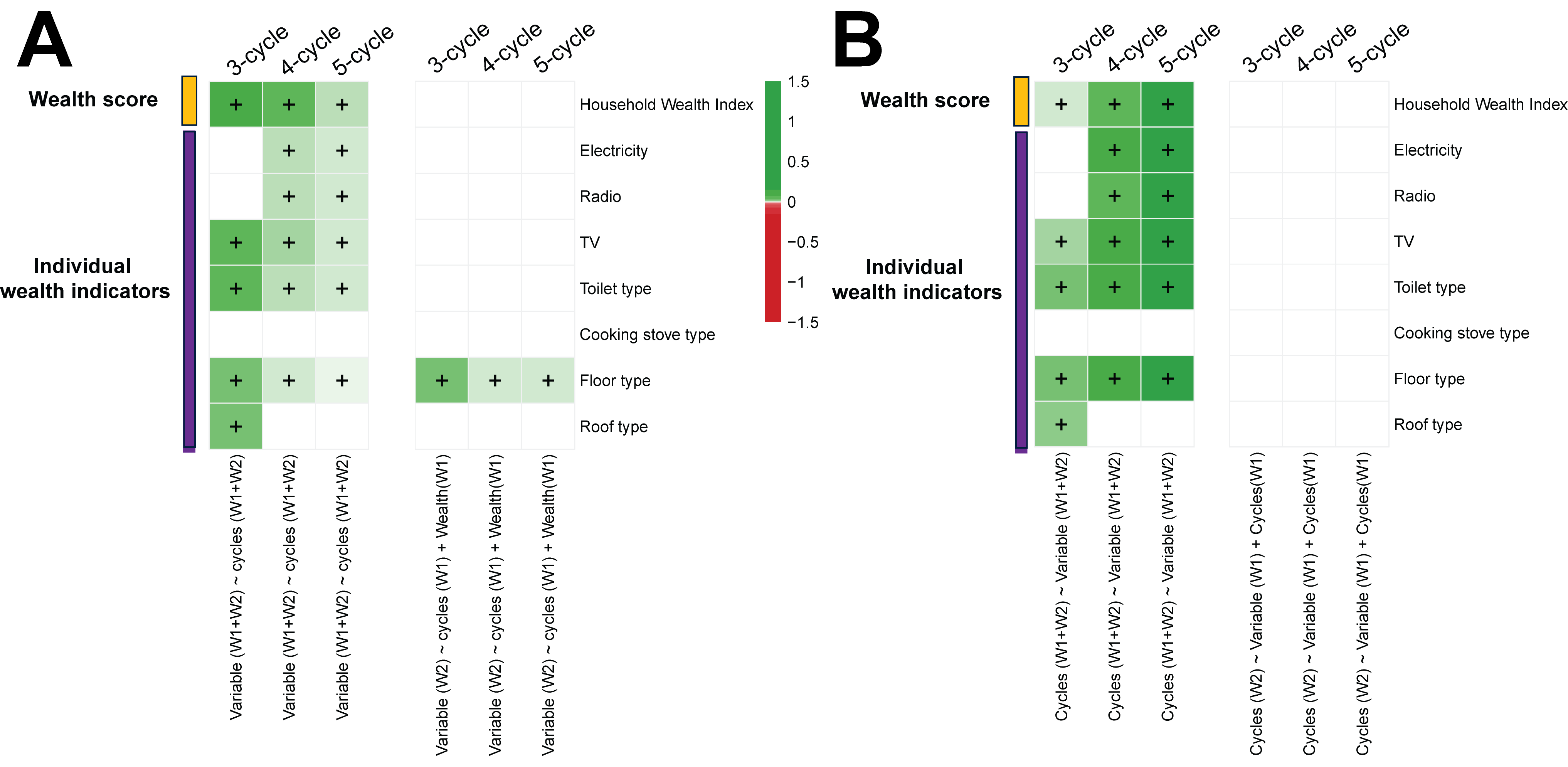


**Figure S4:** Association between wealth variables and cycles (common to both friendship and borrow/lend networks): This shows only thirty-seven significant relationships across both models with wealth and cycles as predictors, respectively. Most of the significant associations are similar to associations in the cycles otherwise originating solely from borrow-lend network ties. Radio and electricity as dependent variables in (A) show more significant associations, whereas cooking stove type and roof type show fewer associations. In addition, temporal regressions with cycles in wave 2 as a dependent variable show no significant associations. All associations shown are adjusted for multiple hypothesis testing using Benjamini-Hochberg correction (FDR<0.05).


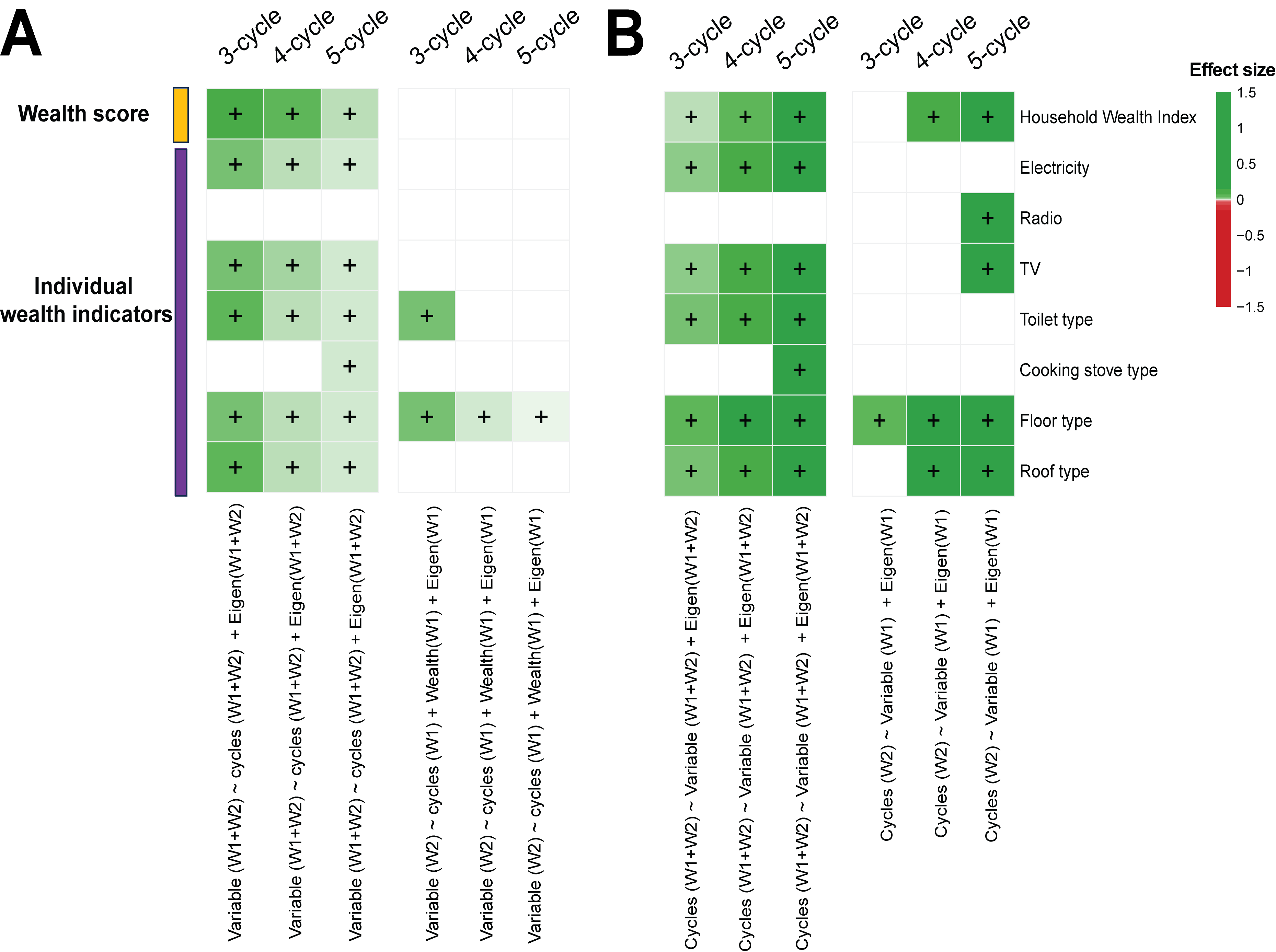


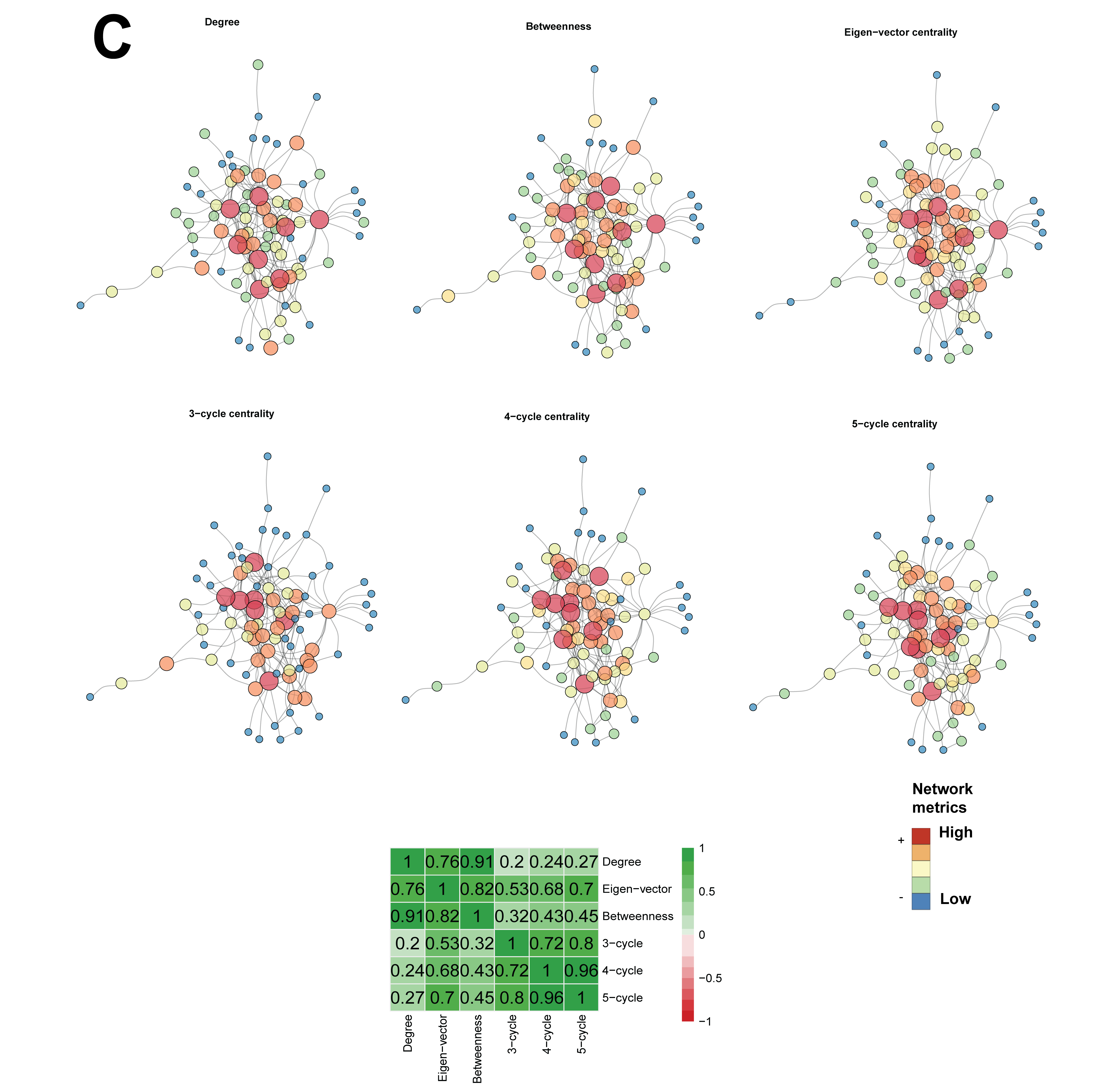


**Figure S5:** Benchmarking our model with eigenvector centrality (added as an additional covariate) and showing relationships with other network metrics: (A and B) This shows fifty-one significant relationships across both models with wealth and cycles as predictors respectively, with wealth variables as predictors (A) and cycles as predictors (B). Most of the significant associations are similar to associations in cycles originating solely from borrow-lend networks (in Figure 2). All associations shown are adjusted for multiple hypothesis testing using Benjamini-Hochberg correction (FDR<0.05). In addition, we show simulated examples of a demonstrative network (C) with 85 nodes and 190 edges showing how similar the network centrality metrics are with cycle centrality metrics.


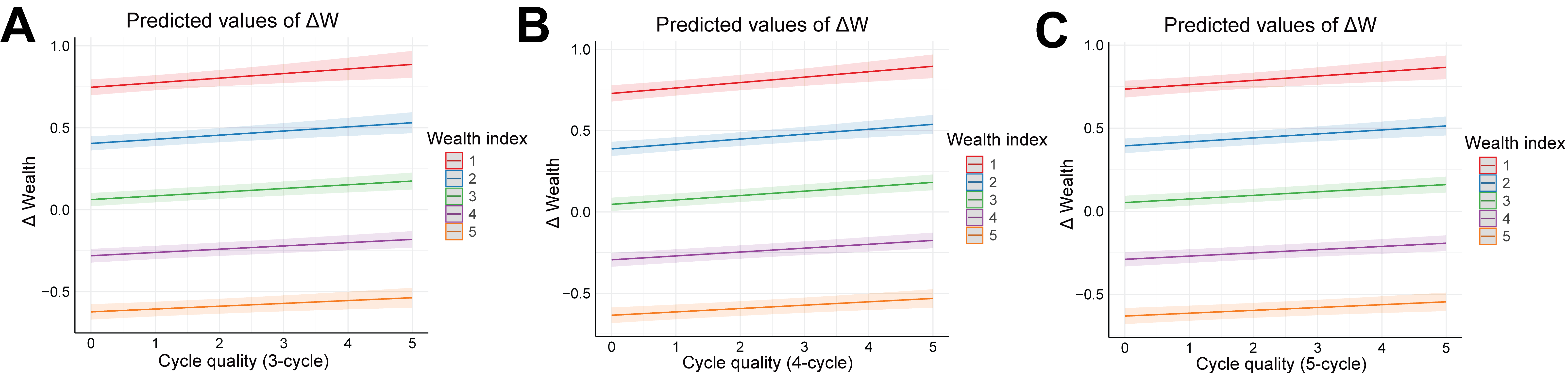


**Figure S6:** Cycle quality and wealth change stratified by wealth class: Response outcomes from 5 different wealth classes on wealth change with cycle quality or composition.


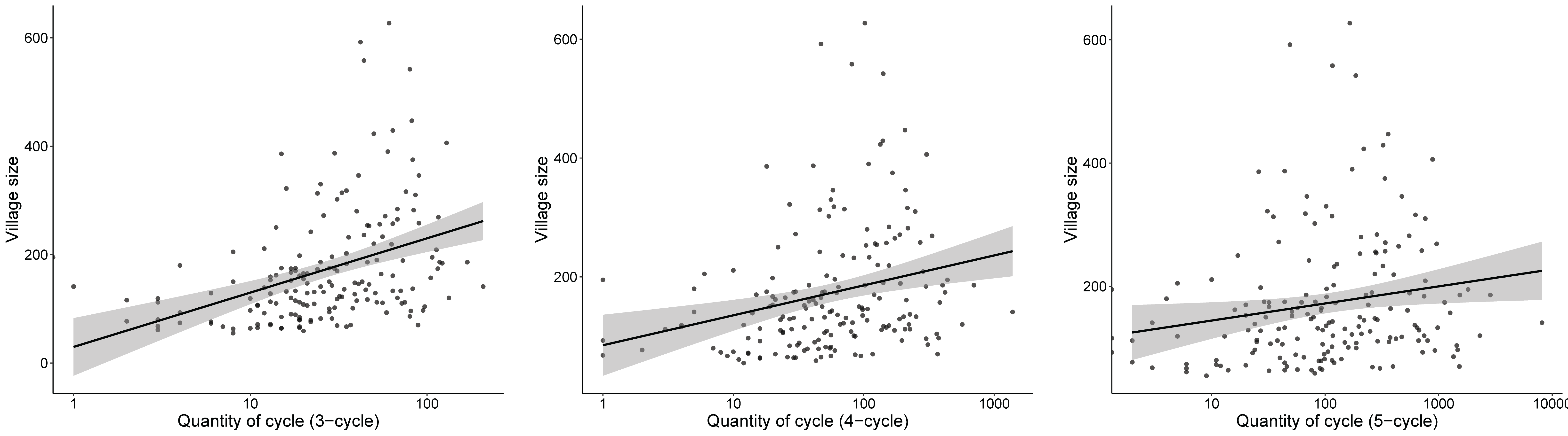


**Figure S7:** Village size and cycle quantity: Illustrative plots showing significant positive association with village size for cycles of length 3 (β=0.9732, p-value = 2.91 x 10^-05^), and no significant associations with cycles of length 4 (β=2.66 x 10^-05^, p-value = 0.636) and 5 (β=2.2 x 10^-04^, p-value = 0.055).


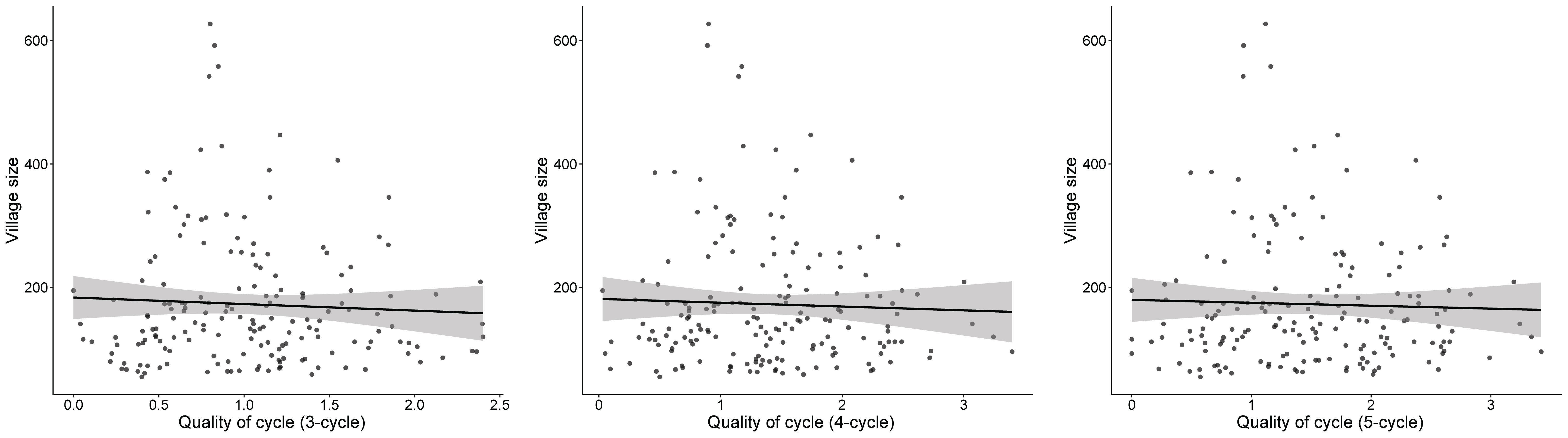


**Figure S8:** Village size and cycle quality: Illustrative plots showing no significant association between village richness and cycle quality of lengths 3 (β= -10.616, p-value = 0.487), 4 (β= -6.18, p-value = 0.6014), and 5 (β= -4.737, p-value = 0.6624).


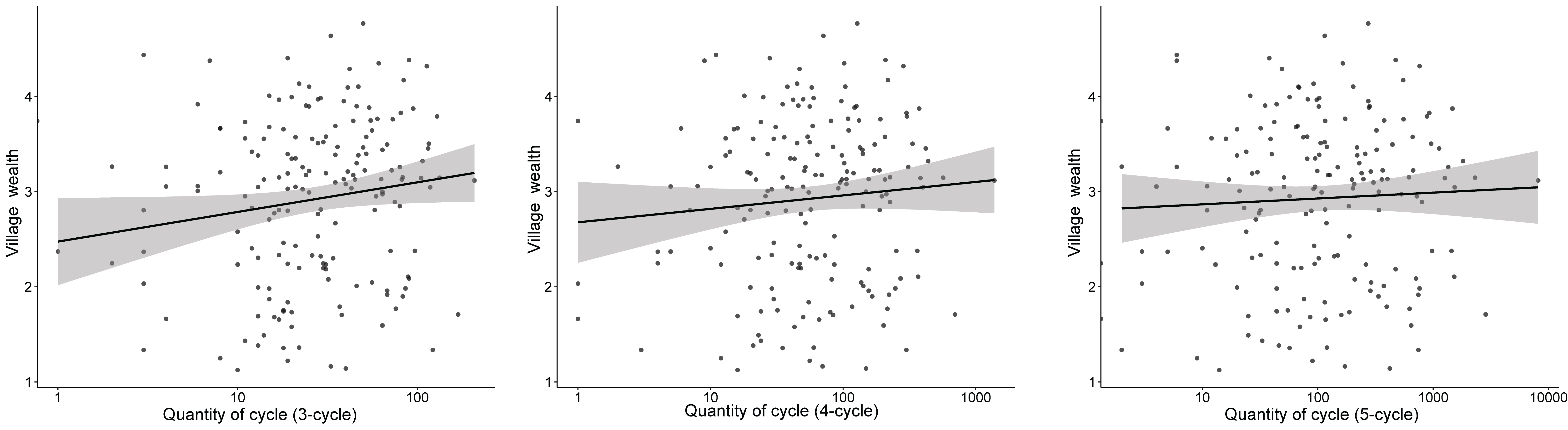


**Figure S9:** Village wealth and cycle quantity: Illustrative plots showing no significant association between village richness and cycle quantity. The results were non-significant for all cycle lengths: 3 (β= -5.63 x 10^-05^, p-value=0.98), 4 (β= -1.49 x 10^-05^, p-value=0.97), 5 (β= 1.01 x 10^-05^, p-value=0.91).


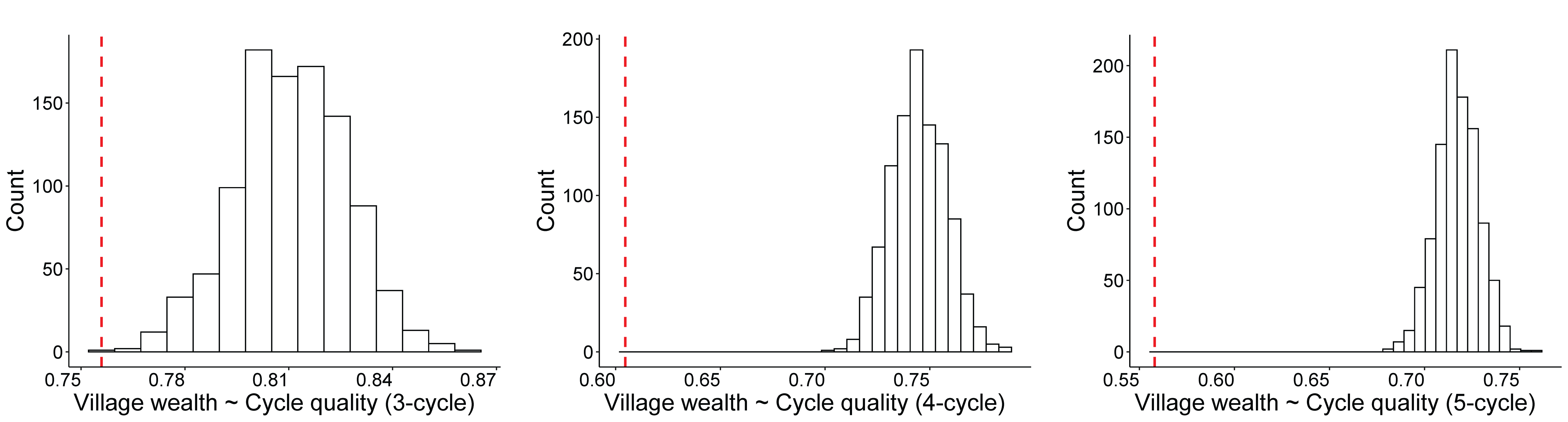


**Figure S10:** Observed versus expected effects of cycle quality on village wealth: Distribution of coefficients (β) from ensemble of 1000 regressions (Village wealth ~ Cycle quality) across 174 randomized villages (with same size and network structure as the real villages). Red vertical dotted line indicates β from real villages. This process was repeated for all cycle lengths (lengths ∈ {3,4,5}). This plot demonstrates that observed β is different from β emerging from expected or random villages (p-value<10^-220^).


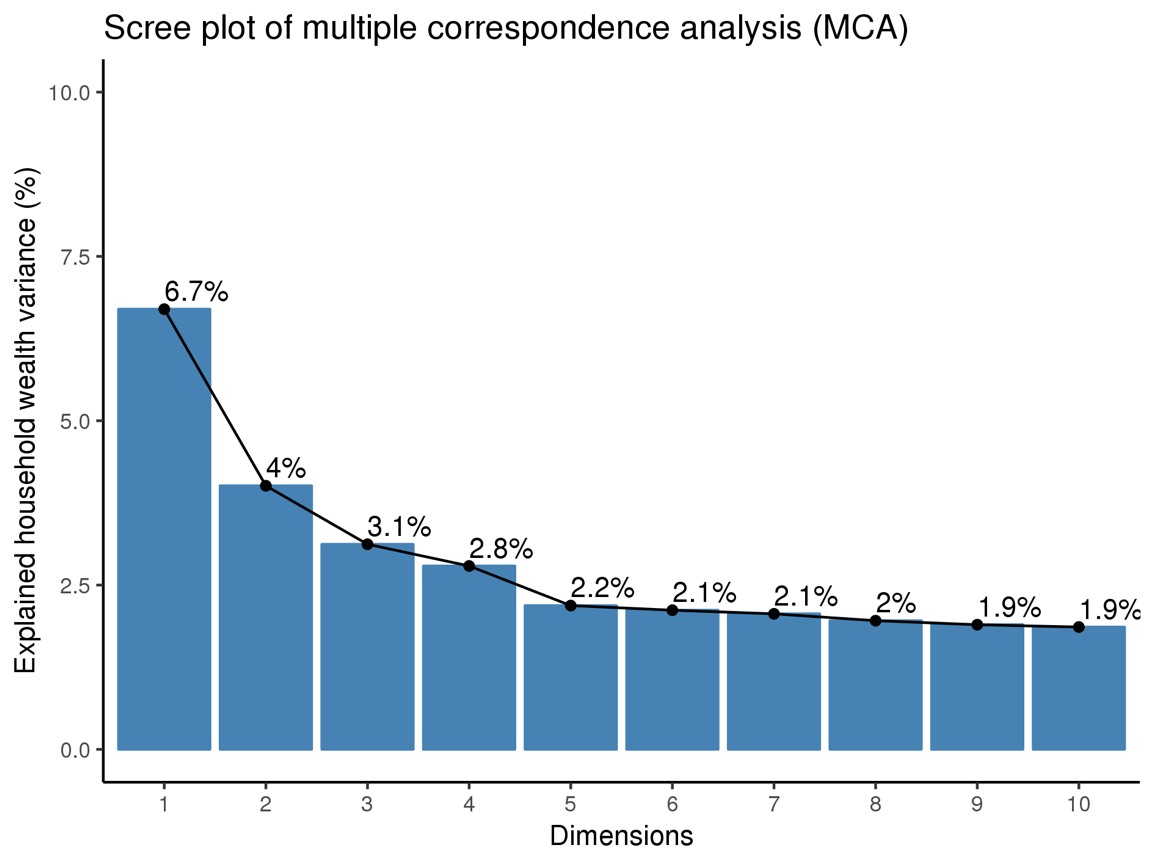


**Figure S11:** MCA components: Amount of variance explained by first 10 dimensions of the MCA (Multiple Correspondence Analysis.


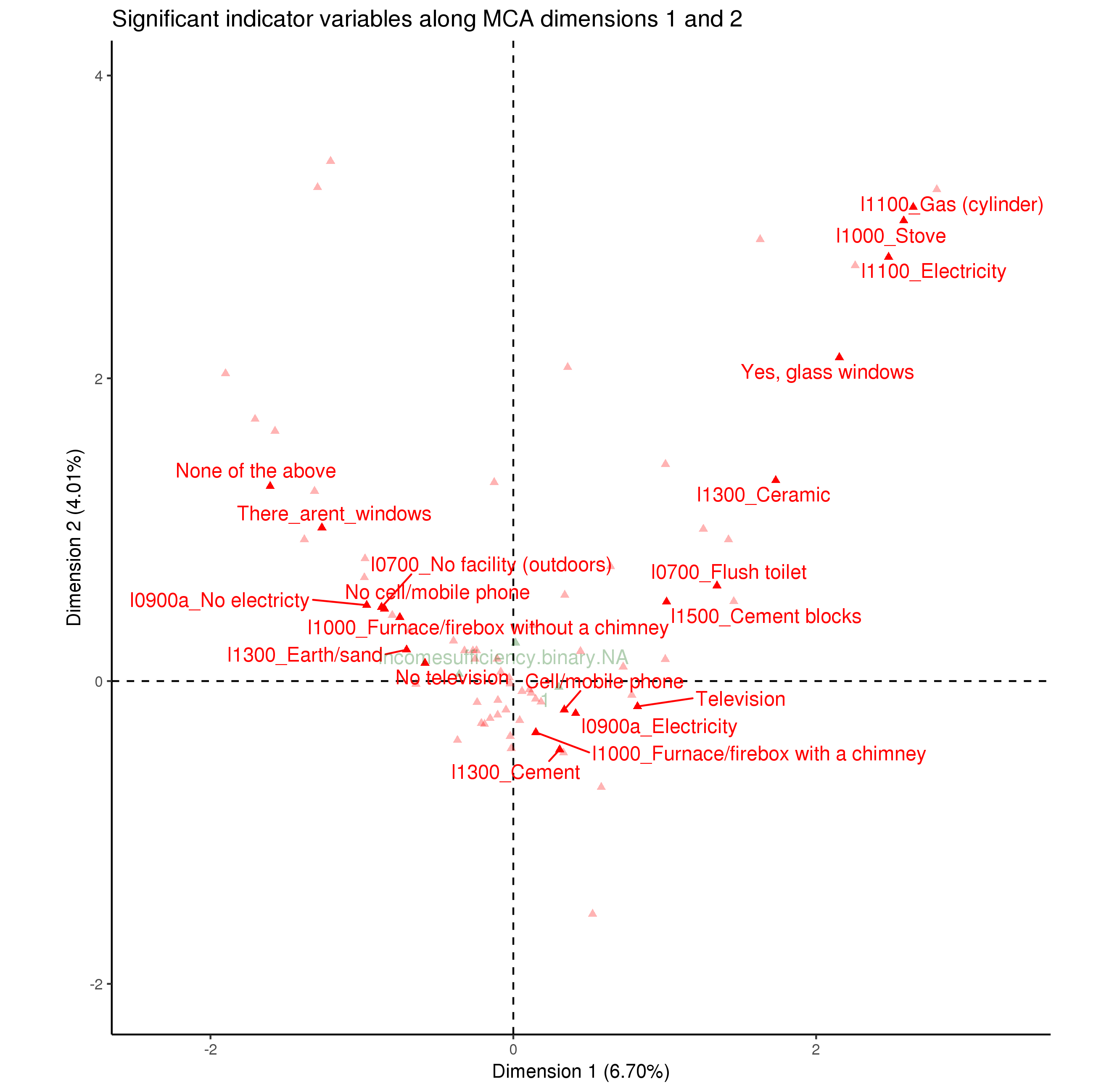


**Figure S12**: Multiple Correspondence Analysis of all wealth variables for first two components: Items with significant contributions to the analysis are labelled on the graph. This plot shows the first two dimensions of the MCA.


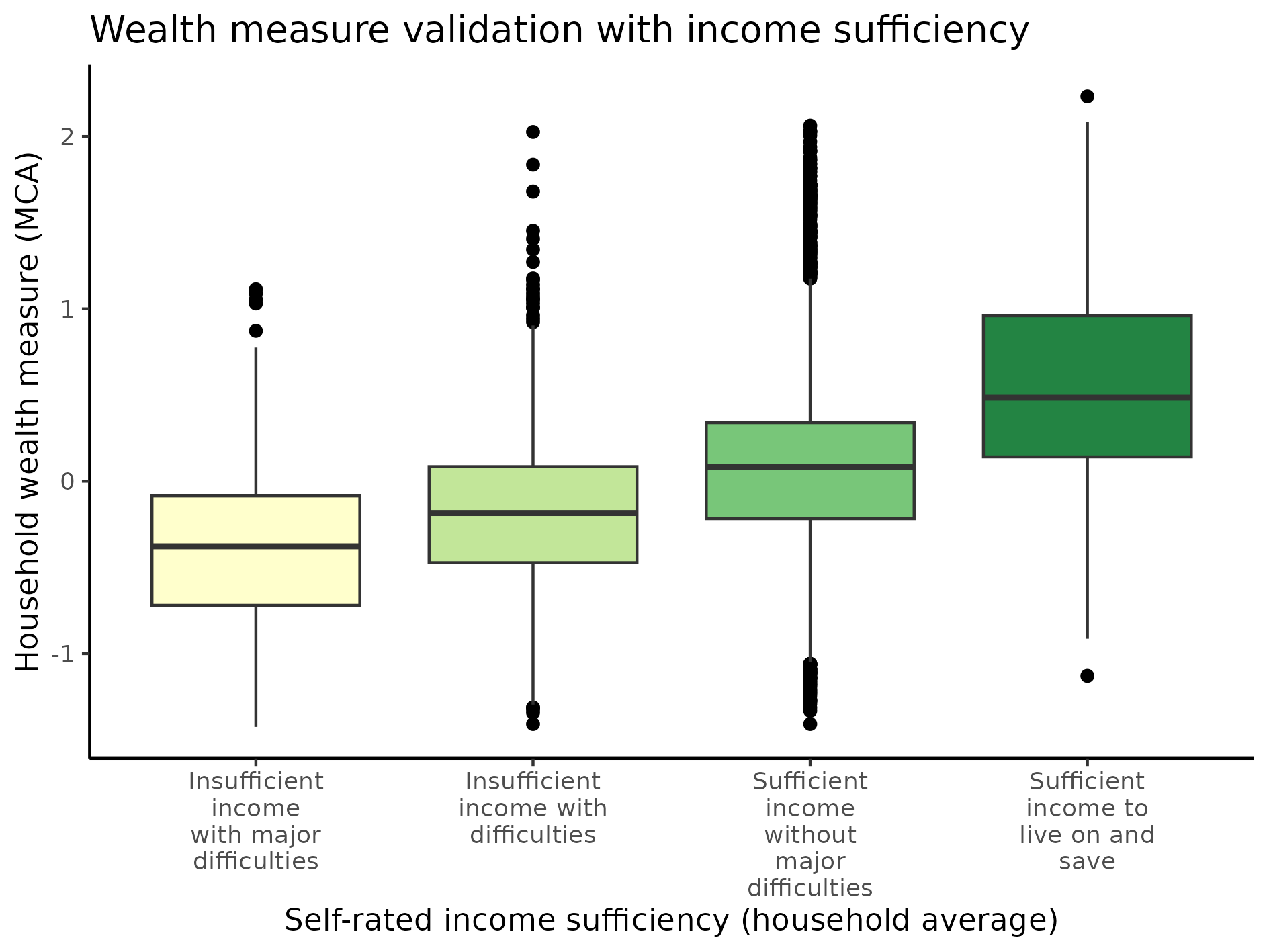


**Figure S13**: Validation of the generated household wealth index: Individuals were surveyed on the sufficiency of their income to meet basic needs using the Food Security Index. These responses were averaged by household and used as a validity check for the MCA-generated household wealth index.


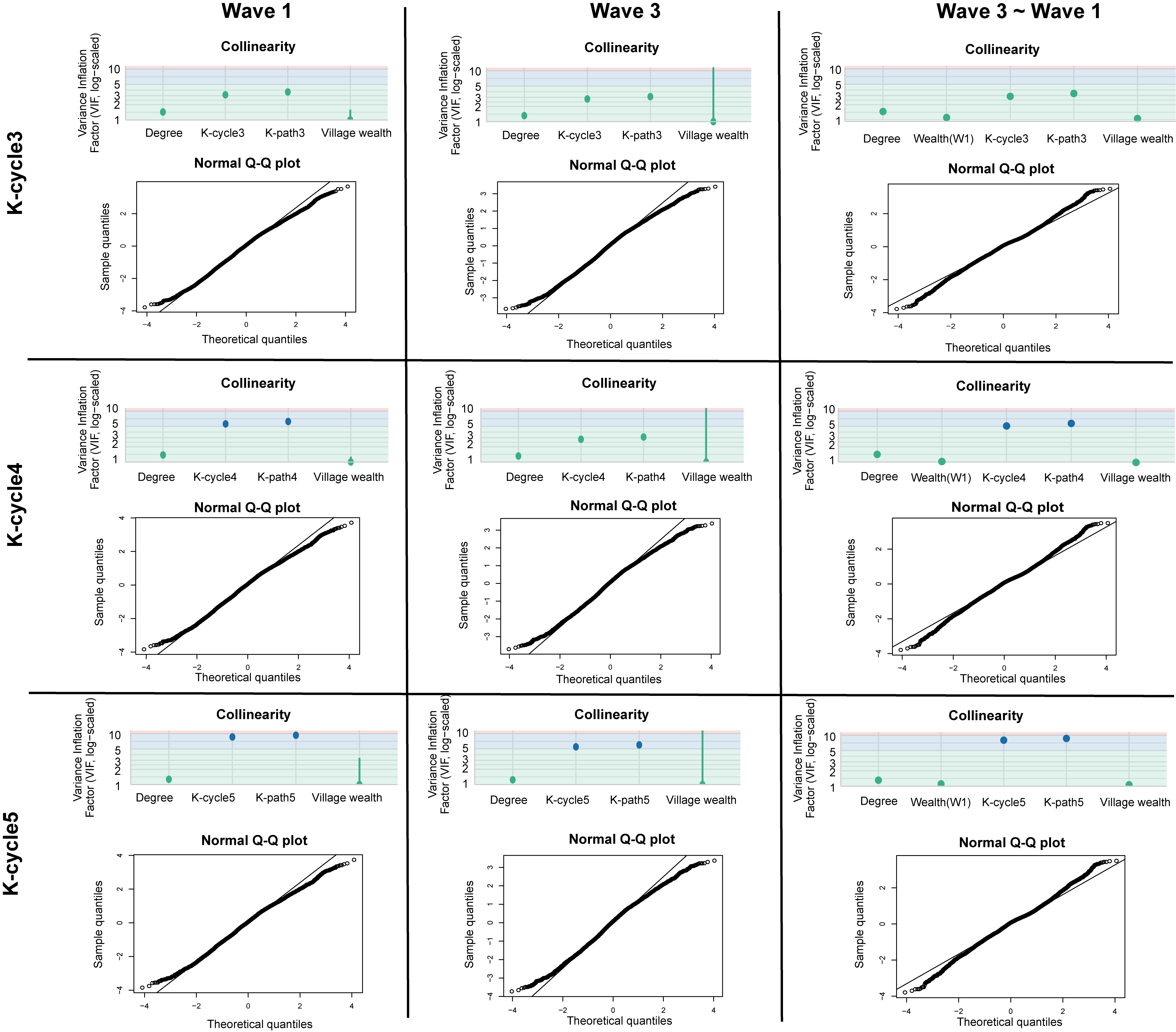


**Figure S14:** Model characteristics for regressions on wealth: Collinearity plots demonstrate the measure of multicollinearity through variance inflation of all independent variables in the model. All values below red line show absence of multicollinearity in the models. Normal Q-Q plots illustrate the measure of heteroskedasticity in the regression models. All Normal Q-Q plots are indicative of homoskedasticity (values close to the diagonal line), making underlying assumption of normalized errors in the regression models to be valid.
